# Minutes of the Organization, Constitution, and Ethics, of the Union Medical Society of Northern Indiana

**Published:** 1845-10

**Authors:** 


### BIBLIOGRAPHICAL NOTICES.

*Minutes of the Organization, Constitution, and Ethics of the Union Medical Society of Northern Indiana.*

This pamphlet contains the minutes of the proceedings of a meeting of the physicians of the counties of Elkhart and Koskiusko, convened at Goshen, Ind., on February 18th, 1845, for the purpose of organizing a Medical Society; also of an adjourned meeting held on April 22d, to complete the organization. At the former meeting, it was unanimously agreed to form an association, to be called the Union Medical Society of Northern Indiana. The officers were elected for one year: a committee appointed to prepare a constitution and bye-laws, a system of ethics, and a bill of rates. The meeting then adjourned until April 22d.

At the adjourned meeting, the constitution and code of ethics were adopted, and the bill of rates further referred to a special committee. Committees were then appointed on the following subjects: "On Quackery," "On Improvement in the Science of Medicine," "On Collateral Sciences." Dr. S. B. Kyler and John Gildersleeve, were appointed to deliver dissertations at the next meeting. "The meeting then adjourned to the second Saturday in October next, to meet in Goshen.

We are glad to see this movement on the part of our professional neighbors in Indiana. We hope that the example will be followed in other sections of that State, and also of Illinois. In the latter, we are not aware of the existence of a single medical society. If any such exist, we would be pleased to hear of it. There can be little doubt but that the influence exerted by medical societies, when conducted in proper spirit, and under proper regulations, is in every way beneficial to the profession and the public, and in our opinion, more preservative of the interests of all parties than can be expected from any medical legislation. It is scarcely to be expected that the wants of a profession, and the measures required for its elevation and to preserve its dignity, should be understood by any but its own members. Coöperation among medical men, in a strict adherence to the courtesies of the profession; the universal adoption of a rigid system of medical ethics; exclusion from consultation, and from social intercourse, of all pretenders; and a rigid scrutiny into the qualifications of all admitted to its privileges, are the means to be adopted for rescuing the science from the imputations against it, and to place it in the estimation of the public, in its proper light.—Ed.

